# Selective enhancement of cost sensitivity by methylphenidate in adult ADHD: a randomized placebo-controlled trial

**DOI:** 10.1017/S0033291726104954

**Published:** 2026-07-28

**Authors:** Mads Lund Pedersen, Athanasia M. Mowinckel, Sigurd Ziegler, Mats Fredriksen, Atle Bjørnerud, Tor Endestad, Guido Biele

**Affiliations:** 1 Department of Psychology, University of Oslo, Oslo, Norway; 2 Center for Precision Psychiatry, Division of Mental Health and Addiction, Oslo University Hospital, Oslo, Norway; 3 Center for Lifespan Changes in Brain and Cognition, University of Oslo, Oslo, Norway; 4 Department of Neurology, Oslo University Hospital, Oslo, Norway; 5 Division of Mental Health and Addiction, Vestfold Hospital Trust, Tønsberg, Norway; 6 Department of Physics, University of Oslo, Oslo, Norway; 7 Unit for Computational Radiology and Artificial Intelligence, Oslo University Hospital, Oslo, Norway; 8 Department of Child Health and Development, Norwegian Institute of Public Health, Oslo, Norway

**Keywords:** ADHD, computational psychiatry, decision-making, drift diffusion model, evidence accumulation, methylphenidate, computational modeling, drift rate, decision threshold, speed-accuracy tradeoff, dopamine, stimulant medication

## Abstract

**Background:**

Attention-deficit hyperactivity disorder (ADHD) is associated with impairments in real-life decisions, many involving balancing costs and benefits. Increasing dopamine and norepinephrine with stimulant medication improves decision-making in ADHD. However, it is unclear whether stimulant medication affects sensitivity to costs and/or benefits or alters the speed-accuracy tradeoff. Here, we applied the drift diffusion model (DDM) to assess how the stimulant medication methylphenidate (MPH) affects subprocesses of cost-benefit decision-making in ADHD.

**Methods:**

Sixty-one adult participants with ADHD and 63 healthy controls completed a cost-benefit decision-making task in a randomized double-blind placebo-controlled design. ADHD participants were tested once on and once off MPH. Control participants performed the task in two sessions without medication. The task entailed deciding whether to accept or reject a stimulus based on learned cost and benefit value associations. The effect of ADHD and medication on subprocesses of decision-making was disentangled by estimating task performance with DDM parameters drift rate, starting point bias, and decision threshold, representing evidence accumulation, a priori accept/reject bias, and the speed-accuracy tradeoff, respectively. Primary analyses used a modified intention-to-treat sample, including participants with main-phase data from both sessions.

**Results:**

The DDM analysis identified impaired evidence accumulation combined with premature responding in participants with ADHD, indicated by reduced sensitivity to costs and benefits and narrower decision thresholds. MPH altered the cost-benefit balance by improving sensitivity to costs. Lastly, lower cost sensitivity was associated with greater symptom severity and higher prescribed dosage in ADHD.

**Conclusions:**

Methylphenidate alters cost-benefit decision-making in ADHD by increasing sensitivity to costs.

## Introduction

Attention-deficit hyperactivity disorder (ADHD) is a neurodevelopmental disorder characterized by high levels of hyperactivity, impulsivity, and/or inattention (American Psychiatric Association, 2013). The precise etiology of ADHD has not been identified, but prevailing neurobiological ADHD theories assume that cortico-striatal dysfunction of the catecholamines dopamine (DA) and norepinephrine (NE) alters cognitive functioning and reward processing (Frank, Santamaria, O’Reilly, & Willcutt, 2007; Sagvolden, Johansen, Aase, & Russell, 2005; Sonuga-Barke, 2003; Tripp & Wickens, 2009), which in turn may cause ADHD symptoms. The central role of catecholamines in theories of ADHD is driven by the fact that ADHD symptoms and associated cognitive impairments are ameliorated with methylphenidate (MPH) (Coghill et al., 2014; Fredriksen & Peleikis, 2016), a central stimulant that increases synaptic availability of DA and NE (Del Campo, Chamberlain, Sahakian, & Robbins, 2011; Volkow, Wang, Fowler, & Ding, 2005).

ADHD is associated with impaired real-life decisions, including reckless driving, substance abuse, and risky sexual behavior (Barkley, Murphy, Dupaul, & Bush, 2002; Faregh & Derevensky, 2011; Flory et al., 2006; Lee et al., 2011; Molina & Pelham, 2003; Sarver, McCart, Sheidow, & Letourneau, 2014). An important feature of most real-life decisions is the need to balance costs and benefits to determine the net value of actions. Upgrading to the newest phone is rewarding but comes with the cost of having less money for other expenses. It is unclear how participants with ADHD deviate from the normal population in balancing costs and benefits. Here we set out to investigate the mechanisms underlying cost-benefit decision-making in ADHD, and how these processes are influenced by MPH.

**Figure 1.**
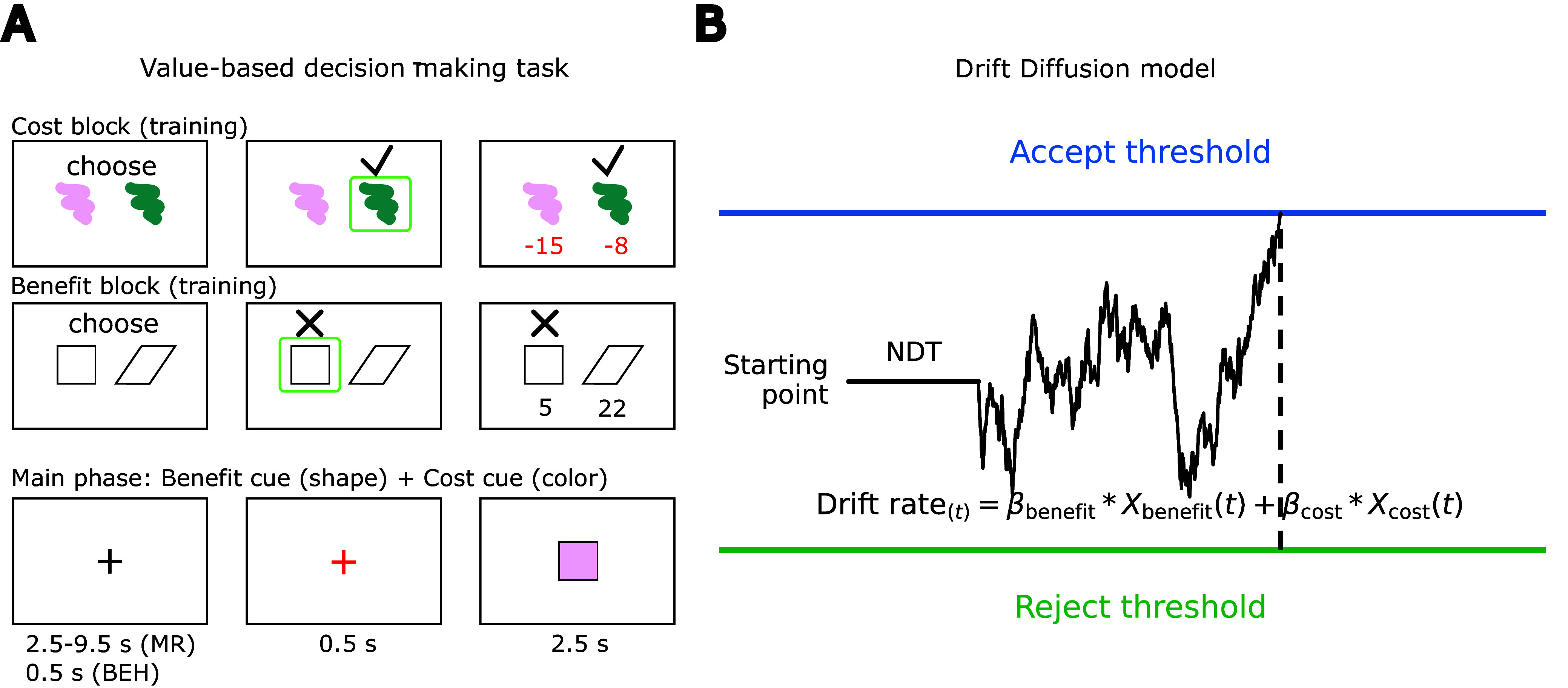
(a). Learning and test phase of the cost-benefit decision-making task. Participants had to learn the benefit and cost values associated with six shapes and six colors stimuli, respectively (or vice versa). During the test phase, participants either accepted or rejected colored shape stimuli, dependent on whether they believed the overall value was positive or negative. (b). Graphical description of the drift diffusion model. Accumulation of evidence begins at the Starting point. The example sample path represents the accumulation of noisy evidence, which is collected until a decision threshold is reached (upper or lower threshold) and a response is initiated. The nondecision time parameter (NDT) accounts for time spent on sensory encoding and motor execution. In the current task, the upper and lower boundaries represented decisions to accept or reject, respectively, stimuli with a combined benefit and cost value. Trial-specific drift rates were modeled as the combination of these values and their estimated slopes. *Note*: MRI, magnetic resonance imaging cohort of the study; BEH, behavioral cohort of the study. Figure 1. long description.

There are several ways in which alterations in underlying cognitive processes could cause alterations in observed cost-benefit decision-making, including altered sensitivity to costs and/or benefits, or in the tradeoff between making fast but more erratic versus slow and more accurate decisions. Computational models can be used to identify and disentangle the cognitive processes underlying behavior (Kriegeskorte & Douglas, 2018), and further explore how these processes are affected in clinical groups (Huys, Michael Browning, Paulus, & Frank, 2021; Maia, Quentin, & Frank, 2017; Pedersen et al., 2021). Here, we apply the drift diffusion model (DDM) (Ratcliff, 1978; Ratcliff & McKoon, 2008), a computational model of decision-making, to capture the dynamic processes underlying choosing to accept or reject offers based on the associated costs and benefits of alternatives. The drift diffusion model decomposes choice and response times into components that make up a process model of decision-making supported by behavioral (Ratcliff & Smith, 2004) and neurobiological data (Forstmann, Ratcliff, & Wagenmakers, 2016; Gold & Shadlen, 2007; Steinemann et al., 2024). The model explains fast choosing between two alternatives as a process of continuous information accumulation until reaching a response threshold in favor of one of the alternatives (Ratcliff & McKoon, 2008) (Figure 1b). The drift rate captures the speed of evidence accumulation dependent on the quality of available evidence and the decision-makers’ ability, level of attention, and effort. The amount of evidence required to reach a decision is modeled by the decision threshold parameter, representing a tradeoff between speed and accuracy. The intercept of the drift process captures a priori preference for a decision alternative, while time spent on sensory encoding and motor execution is accounted for by the nondecision time parameter. In the current task, participants decided whether to accept or reject a stimulus based on its overall value (Figure 1), which was determined by learned associations between shapes and benefits, and colors and costs (or vice versa, counterbalanced across participants). For this cost-benefit decision-making task, the drift rate on each trial was estimated as a weighted combination of benefit and cost values, while decision thresholds represented choosing to either accept or reject the stimulus. The starting point parameter represented whether participants displayed tendencies to favor accepting or rejecting before seeing the stimulus on the current trial.

We have previously derived specific predictions about the effect of ADHD on DDM parameters from neurobiological theories of ADHD (Ziegler, Pedersen, Mowinckel, & Biele, 2016). Based on assumptions of deficient DA (or both DA and NE) signaling, the theories predict that impaired decision-making in ADHD results from reduced drift rates and narrower decision thresholds (Frank et al., 2007; Sagvolden et al., 2005; Tripp & Wickens, 2009) due to inefficient accumulation of evidence and responding before having accumulated sufficient information. Meta-analyses support the prediction of drift rate reduction in ADHD, as both children (Karalunas et al., 2014) and adults (Mowinckel, Pedersen, Eilertsen, & Biele, 2015) show lower drift rates relative to peers. Further, studies have found that MPH improves information accumulation, that is, drift rate, in perceptual tasks for children with ADHD (Fosco, White, & Hawk, 2017) and healthy controls (Beste et al., 2018). It is less straightforward to deduce predictions on the implications of ADHD and central stimulants on the balancing of costs and benefits. DA increases tendencies to learn from positive compared to negative reinforcements (Frank, Seeberger, & O’Reilly, 2004) and is also predicted to increase focus on positive compared to negative values during choice (Collins & Frank, 2014). However, ADHD is associated with decreased sensitivity to losses in temporal discounting and gambling (Luman, Oosterlaan, Knol, & Sergeant, 2008; Tanaka et al., 2018). The observed effects of ADHD on decision thresholds do not consistently support the prediction of narrower decision thresholds. Although one study reported impaired speed-accuracy adjustment in ADHD (Mulder et al., 2010), results from meta-analyses are inconclusive as to whether participants with ADHD make decisions based on less information than their healthy peers (Karalunas et al., 2014; Mowinckel et al., 2015). Studies assessing the effect of MPH on decision parameters have reported a reduction of decision threshold following stimulant medication (Fosco et al., 2017; Loughnane et al., 2019; Weigard, Heathcote, & Sripada, 2019), which, accompanied by improved evidence accumulation, has been interpreted as an improved ability to optimize decision thresholds.

Here, we examined whether adults with ADHD show reduced evidence accumulation, altered response caution, and altered cost–benefit weighting compared with healthy controls, and whether methylphenidate normalizes these parameters. We hypothesized that (a) ADHD would be characterized by reduced evidence accumulation, operationalized as lower drift-rate sensitivity to both benefits and costs, and by premature responding reflected in a lower decision threshold. We further hypothesized that (b) methylphenidate would increase evidence accumulation by increasing drift-rate sensitivity to value information. Given mixed prior evidence on valence-specific effects, we tested whether methylphenidate effects on drift-rate sensitivity differed for costs versus benefits.

## Methods and materials

The study was approved by the Regional Committee of Medical Health Research Ethics (South-East Norway; Project IDs: 2011/1585 and 2012/1105), and the Norwegian Medicines Agency (EudraCT: 2012-005246-38). Informed written consent was obtained from all participants.

Participants enrolled in an MRI- or behavioral cohort and participated in two testing sessions. Participants in the MRI cohort performed the task in an MR scanner at the Oslo University Hospital. Participants in the behavioral cohort were tested in a quiet room at the University of Oslo, or in the outpatient clinic at Vestfold Hospital, depending on participants’ preference. The testing procedure lasted ~2.5 hours in the MRI cohort and 2 hours in the behavioral cohort. Participants were paid between 350 and 500 NOK (ca. 35–50 USD) each session, depending on task performance. The research was carried out in compliance with the Helsinki Declaration.

### Participants

Participants had to be between 18 and 40 years old and have normal or corrected-to-normal vision. Exclusion criteria included history of drug or alcohol abuse, severe psychiatric comorbidity, and current medication with psychopharmacological agents for other disorders than ADHD (see Supplementary Material S1 for full list of exclusion criteria). Participants in the MRI cohort were screened for contraindications for MRI via telephone and at each session before test commencement, using the hospital protocol for MRI research participation.

#### ADHD participants

Participants diagnosed with ADHD were recruited through an outpatient clinic at Vestfold Hospital Trust, Norway. All patient participants were diagnosed and currently receiving care at the clinic (see Supplementary Material S2 for diagnostic procedures) and taking methylphenidate (in the form of Ritalin™ extended-release capsules or immediate-release tablets). Sixty-one adults with ADHD were recruited to the study (see Supplementary Figure S1 for Consort flow diagram and Supplementary Material S4 for dates of first and last recruitment). Neuroimaging data from the MRI cohort is published in a separate article (Mowinckel et al., 2017).

#### Controls

Three thousand men and women living in Oslo were randomly selected by Statistics Norway to receive an invitation via mail to participate in the research project as control participants. Among the 372 responders, we contacted people matching ADHD participants on age and sex, and as close as possible on education. Control participants were screened with the Adult ADHD self-report scale via telephone and were excluded if they scored 15 or higher on the first 6 items. At inclusion, 33 control participants were recruited to the MRI study and 30 to the behavior study.

The healthy control sample was not fully matched to the participants on education and WAIS scores (Table 1). Because lower education and reduced scores on tests of intelligence are consequences of ADHD, fully matching these variables would likely remove decision-making effects of ADHD mediated by education and intelligence, leading to a biased estimate of effects of ADHD on decision-making (de Zeeuw et al., 2017).

**Table 1.** Sample characteristics Table 1. long description.

	ADHD		Controls	
*N* (male/female)	57 (20/37)		60 (19/41)	

	*M*	SD	*M*	SD
Education (y)	12	1.8	14.7	2.2
Age (y)	27.9	6	27.4	5.7
ASRS	12.3	4.9	6.9	2.9
WAIS similarities	10.3	3.4	12.9	3
WAIS matrices	10.7	3.5	12.6	3.1
Test interval (d)	29.6	12.2	28.4	10.5

*Note*: ASRS, Adult ADHD Self-Report Scales; WAIS, Wechsler Adult Intelligence Scale; *M*, mean; SD, standard deviation; y, years; d, days.

**Table 2.** Summary behavioral and modeling results (mITT sample) Table 2. long description.

Parameter	HC	MPH	PLC	HC vs. PLC	HC vs. MPH	MPH vs. PLC
Accuracy	0.91 [0.90, 0.93]	0.86 [0.83, 0.88]	0.85 [0.82, 0.87]	0.07 [0.04, 0.10]	0.06 [0.03, 0.09]	0.01 [−0.01, 0.03]
Mean RT	1.02 [0.97, 1.07]	0.95 [0.90, 0.99]	0.97 [0.92, 1.03]	0.05 [−0.02, 0.12]	0.07 [0.00, 0.14]	−0.02 [−0.07, 0.01]
SD RT	0.36 [0.34, 0.37]	0.33 [0.31, 0.35]	0.36 [0.34, 0.38]	−0.00 [−0.03, 0.02]	0.03 [0.00, 0.05]	−0.03 [−0.05, −0.01]
Starting point bias	0.53 [0.52, 0.55]	0.51 [0.50, 0.53]	0.51 [0.50, 0.53]	0.02 [0.00, 0.04]	0.02 [−0.00, 0.04]	0.00 [−0.00, 0.01]
Decision threshold	2.08 [2.00, 2.17]	1.87 [1.79, 1.96]	1.91 [1.82, 2.00]	0.18 [0.05, 0.30]	0.21 [0.09, 0.33]	−0.03 [−0.05, −0.01]
Non-decision time	0.42 [0.40, 0.44]	0.39 [0.37, 0.41]	0.37 [0.35, 0.39]	0.05 [0.02, 0.08]	0.03 [0.00, 0.06]	0.02 [0.01, 0.02]
Cost sensitivity	1.02 [0.93, 1.12]	0.85 [0.74, 0.95]	0.65 [0.55, 0.75]	0.37 [0.24, 0.52]	0.18 [0.04, 0.32]	0.20 [0.17, 0.23]
Benefit sensitivity	0.98 [0.90, 1.07]	0.73 [0.65, 0.82]	0.71 [0.62, 0.80]	0.27 [0.15, 0.39]	0.25 [0.13, 0.37]	0.02 [−0.01, 0.05]
Drift rate intercept	0.07 [−0.00, 0.14]	−0.00 [−0.08, 0.08]	−0.01 [−0.09, 0.07]	0.08 [−0.02, 0.19]	0.08 [−0.03, 0.18]	0.01 [−0.03, 0.05]

*Note:* HC, healthy control; MPH, methylphenidate; PLC, placebo. Mean and 95% credible intervals of posterior distributions.

Four participants were lost to follow-up (two ADHD and two HCs) and did not complete the second session. In addition, two participants had incomplete behavioral data due to technical/recording difficulties (one ADHD and one HC), with the ADHD participant missing main-phase data from session 2. One ADHD participant failed to meet the training criterion. One ADHD participant scored below the predefined WAIS criterion (combined scaled score < 4) and did not return for the second session.

The primary analyses were conducted in a modified intention-to-treat sample (mITT; participants with main-phase behavioral data from both sessions), comprising 57 ADHD participants and 60 healthy controls (Table 1). We additionally report an adherence-filtered sensitivity analysis excluding four ADHD participants with elevated ritalinic acid in the placebo condition (> 1,000 nM; see Supplementary Material S5) and the participant who failed training, resulting in 52 ADHD participants and 60 healthy controls.

### Task

Participants performed a two-alternative value-based decision-making task (Basten, Biele, Heekeren, & Fiebach, 2010) (Figure 1a). During the training phase, which directly preceded the main task, participants learned to associate six shapes with different positive values (benefits) and six colors with different negative values (costs), or vice versa, in separate blocks. The training phase consisted of a minimum of 4 blocks of 45 trials each. During the training phase, participants learned stimulus–value associations through two-alternative choices. On each trial, two stimuli were presented, and an instruction indicated the block rule (‘choose the largest benefit’ or ‘choose the smallest cost’) (Supplementary Figure S2). Numeric values for both stimuli were provided as feedback following each response. Outcomes were deterministic, but values presented after a choice were drawn from nonoverlapping uniform distributions to make it more demanding. Participants with running accuracy below 95% at the end of block 4 completed two additional blocks to ensure that associations were learnt. Training trials were used for familiarization only and were not included in any analyses of main-phase performance or in the DDM fitting.

In the main phase of the task, participants were instructed to use learnt benefit values associated with shapes and cost values associated with colors (or vice versa), and decide to accept or reject a colored shape if the net value (benefit–cost) of a colored shape was positive or negative, respectively. Combinations of stimuli that yielded a net value of zero were not presented, meaning there was always an accurate response. For clarity, we refer to the stimulus feature conveying benefit value as a ‘benefit cue’ and the feature conveying cost value as a ‘cost cue’. The main phase consisted of 176 (MRI cohort) or 420 (behavioral cohort) trials completed over two blocks of equal length. Fewer trials were used in the MRI cohort due to the longer inter-trial stimulus duration required to estimate event-related blood oxygenation level dependent signal. The test phase lasted about 25 minutes in both cohorts. A trial started with a fixation cross, presented for a total of 3–10s in the MRI cohort, and 1s in the behavioral cohort. The fixation cross changed from white to red 0.5 s before stimulus presentation to notify of the upcoming trial. Choice stimuli were presented for 2.5s in the center of the screen. Responses could be made during the entire stimulus presentation. A white fixation cross was presented from the time a response was made until the end of the 2.5-s stimulus presentation period. All participants were instructed to focus on being as fast and accurate as possible, and were informed that they would be compensated based on accuracy in the test phase.

### Procedure

Following a double-blinded crossover procedure, ADHD participants were administered MPH and placebo in randomized order (see Supplementary Material S3 for randomization and administration procedure). Healthy control participants were also tested twice, but did not receive MPH or a placebo. The minimum and maximum intervals between sessions were 14 and 40 days, respectively, with an average interval of 29.6 (ADHD) and 28.4 (Healthy controls) days (Table 1). ADHD participants were assigned to one of four possible dose groups, depending on their normal morning intake of MPH: 1×10 mg *instant-release* (IR) tablet, 2×10 mg IR tablet, 1×20 mg *slow-release* (SR) capsule, or 2×20 mg SR capsule.

To minimize acute medication effects during testing, ADHD participants were instructed to abstain from methylphenidate medication and alcohol for at least 20 hours before participation, and from caffeine for 4 hours prior. This interval was chosen because all medicated participants used methylphenidate formulations (Ritalin immediate-release tablets or Ritalin extended-release capsules), for which the adult elimination half-life is short and typically reported in the range of ~2–3.5 hours (Novartis Pharmaceuticals Corporation, 2025; Patrick, Mueller, Gualtieri, & Breese, 1987). Thus, a 20-hour abstinence period corresponds to multiple half-lives and was expected to allow substantial elimination of methylphenidate from both immediate- and extended-release formulations. Participants receiving doses not exactly within the four dose groups were allocated to the group most closely corresponding to their normal dose, after consultation with their psychiatrist, but were never given a dose higher than their prescription.

### Analysis

Choice and response time data from the value-based decision-making task were analyzed in two ways: separately, using accuracy and response time models, and jointly, using the drift diffusion model (DDM). The analyses used hierarchical modeling to better estimate group and individual parameters (Kruschke, 2010). Posterior distributions were estimated through Markov-chain Monte Carlo (MCMC) simulations. Analyses were performed without nonresponse trials (2.0% of all trials) and without responses faster than 0.3 s (0.2% of all trials), that is, fast guesses.

#### Analysis samples

Primary analyses were performed in the modified intention-to-treat (mITT) sample (participants with main-phase behavioral data from both sessions). We additionally report an adherence-filtered sensitivity analysis excluding protocol nonadherence (ritalinic acid >1,000 nM in placebo) and failed training. Individual-differences analyses involving blood measures and prescribed dose were performed in the adherence-filtered sample.

#### Bayesian inference for choices and response times

Choice and response time data from the value-based decision-making task were analyzed with logistic and linear Bayesian hierarchical regression models, respectively, with the brms package (Bürkner, 2017) in R using uninformative priors.

#### Drift diffusion modeling

Choice and response time data from the value-based decision-making task were fit to the DDM using the brms package (Bürkner, 2017) in R. The model was run with weakly informative priors ranging from typical parameter estimates:

$$\mathrm{\mu v}\sim \text{normal}\left(0,2\right)$$

$$\mathrm{\mu a}\sim \text{normal}\left(1.5,1\right)$$

$$\mathrm{\mu z}\sim \text{normal}\left(0.5,0.2\right)$$

$$\text{μndt}\sim \text{normal}\left(0.2,0.1\right)$$

To link trial-wise offer values to decisions, we modeled the drift rate as a linear combination of the benefit value and cost value of the offer on trial *t*:

$$v\left(t\right)=\beta_{\text{benefit}}*{\text{value}}_{\text{benefit}}\left(t\right)+\beta_{\text{cost}}*{\text{value}}_{\text{cost}}\left(t\right)\;$$

where $x_{\text{benefit}}\left(t\right)$ and $x_{\text{cost}}\left(t\right)$ are the benefit and cost cue values on trial t (*z*-scored across trials), and $\beta_{\text{benefit}}$ and $\beta_{\text{cost}}$ quantify sensitivity to benefits and costs, respectively. Positive *v*(*t*) biases accumulation toward accept, whereas negative *v*(*t*) biases accumulation toward reject. Under this parameterization, higher $\beta_{\text{benefit}}>0$ increases the extent to which benefits speed accumulation toward acceptance, while more negative $\beta_{\text{cost}}<0$ increases the extent to which costs speed accumulation toward rejection. For ease of interpretation, we report $|\beta_{\text{cost}}|$ so that larger values indicate greater cost sensitivity.

We estimated group- and individual-level parameters using hierarchical (multilevel) modeling with participant-specific random intercepts (and random slopes for benefit and cost effects on drift rate). Fixed effects captured diagnosis and medication/session effects.

Drift rate:

$$\begin{array}{l} v\sim \text{benefit}*\text{group}*\text{session}+\text{cost}*\text{group}*\text{session}\\ \;+\;\left(1+\text{benefit}+\text{cost}|\mathrm{id}\right) \end{array}$$

Decision threshold, nondecision time, and starting point:

$$a\sim \text{group}*\text{session}+\left(1\;|\;\mathrm{id}\right)$$

$$\mathrm{ndt}\sim \text{group}*\text{session}+\left(1\;|\;\mathrm{id}\right)$$

$$z\sim \text{group}*\text{session}+\left(1\;|\;\mathrm{id}\right)$$

To model the crossover design, the ADHD group indicator reflected treatment order (HC; PLC→MPH; MPH→PLC) and session was dummy-coded (session 1 vs. 2). All posterior distributions were estimated via MCMC. We compared the main DDM specification (separate benefit and cost sensitivities) to a net-value model using PSIS-LOO. Pareto-k diagnostics indicated influential observations and refit-based remedies were unstable for the Wiener likelihood; we therefore interpret the model comparison as approximate and report full diagnostics in Supplementary Table S3.

#### Associations of individual parameters with prescribed dosage and symptom severity

Further, we extracted individual estimates and tested for associations with symptom severity, measured with WURS (Ward et al., 1993), prescribed dosage, and ritalinic acid within the ADHD group. Specifically, we ran Bayesian linear regression models for each parameter (and for the drift rate, the sensitivity to cost and benefit), assessing the association of symptom severity and interaction with medication status:

parameter~wurs∗medication

Parameters and WURS were *z*-scored, and medication was dummy-coded (0 = Placebo, 1 = MPH) before analysis. For the association between parameter estimates and prescribed dosage, we restricted the analysis to estimated parameters from the MPH-condition:

parameter~dosage

Parameters and prescribed medication dosage were *z*-scored before analysis. Finally, we tested for associations between parameter estimates and ritalinic acid in the MPH condition:

parameter~ritalinic acid

Parameters and levels of ritalinic acid were *z*-scored before analysis.

#### Inferential statistics

For inferential statistics, we report the posterior mean and 95% highest density credible intervals (CrIs) to represent the range of most likely values.

## Results

We analyzed the effect of ADHD and ADHD medication on a cost–benefit decision-making task in a randomized placebo-controlled crossover design. Primary analyses are reported for the modified intention-to-treat (mITT) sample (57 ADHD and 60 controls). We additionally report adherence-filtered sensitivity analyses (52 ADHD and 60 controls) in the Supplement unless otherwise stated.

### Training phase

Training phase results from seven participants (three ADHD and four HCs) were lost due to technical difficulties and were not included in analyses on training phase performance, resulting in analyses on 54 ADHD participants and 56 healthy controls. Participants with ADHD made fewer correct choices than controls on the training phase of the task, both on (HC > MPH: Δ = 0.04, 95% credible interval [0.02, 0.07]) and off (HC > PLC: Δ = 0.04 [0.02, 0.07]) medication. Accuracy was not affected by medication in ADHD (MPH > PLC: Δ = −0.0 [−0.02, 0.02]). A higher proportion of ADHD participants compared to controls required additional blocks of training to reach accuracy criteria, both on (HC > MPH: Δ = −0.26 [−1.06, 0.55]) and off (HC > PLC: Δ = −0.35 [−1.16, 0.46]) medication, and this was more prevalent off compared to on medication (MPH > PLC: Δ = −0.09 [−0.92, 0.75]).

### Accuracy and response time

**Figure 2.**
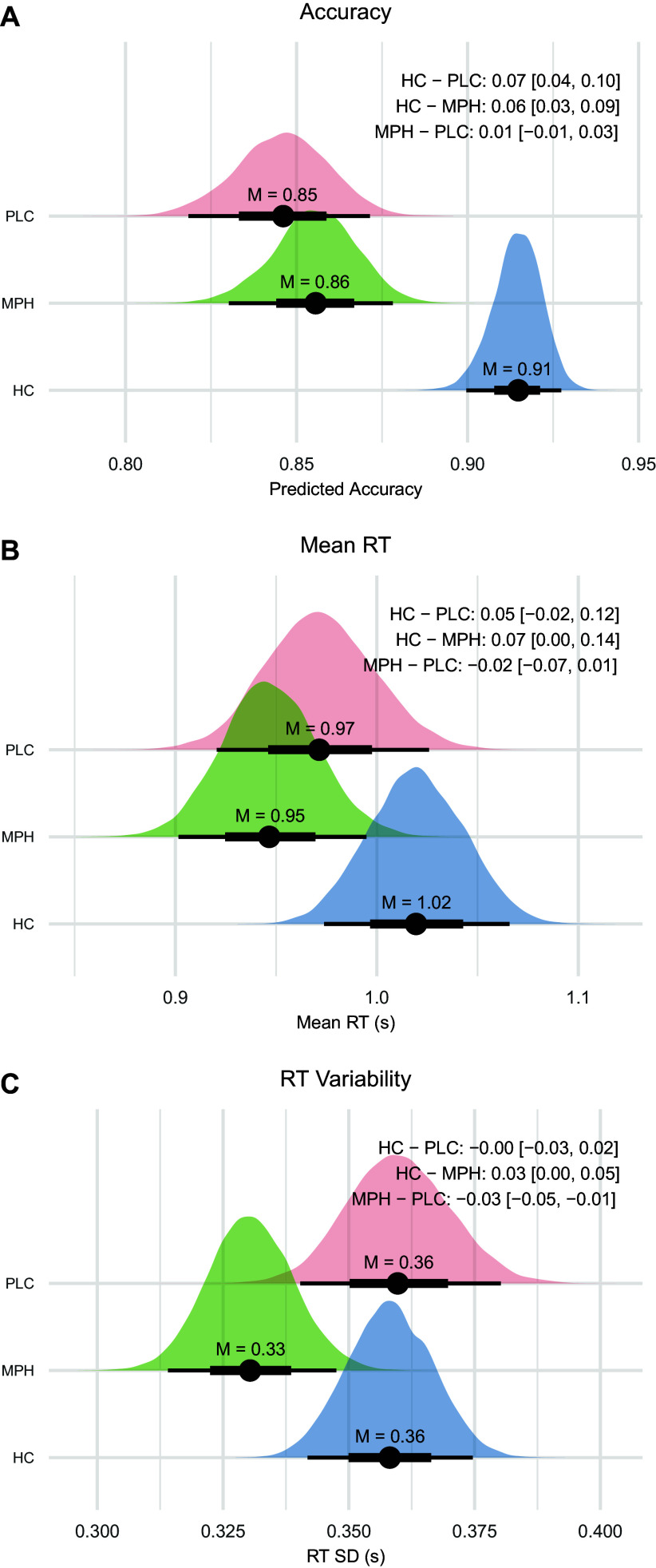
Posterior distributions of choice and response time analyses (mITT sample). Estimated posterior distributions for accuracy (a), response time (b), and response time (RT) variability (c) for each group, with pairwise contrasts reported as posterior mean differences (Δ) with 95% credible intervals (CrIs). Points indicate posterior medians, and thick/thin intervals indicate 66%/95% CrIs. The vertical CrIs include between-participant variation, so that within-comparisons can show clear effects even when CrI intervals for groups overlap. *Note*: HC, healthy controls; MPH, methylphenidate; PLC, placebo; SD, standard deviation. Figure 2. long description.

Confirming our hypothesis, participants with ADHD were less accurate than controls, both on (HC > MPH: Δ = 0.06 [0.03, 0.09]) and off (HC > PLC: Δ = 0.07 [0.04, 0.1]) medication (Figure 2, Table 2). Mean response times were longer in controls compared to participants with ADHD on (HC > MPH: Δ = 0.07 [0.0, 0.14]) and off (HC > PLC: Δ = 0.05 [−0.02, 0.12]) medication. Response time variability did not differ between ADHD on placebo and controls (HC > PLC: Δ = −0.0 [−0.03, 0.02]), while ADHD participants on MPH had less variable response times than controls (HC > MPH: Δ = 0.03 [0.0, 0.05]). Accuracy increased with MPH administration (MPH > PLC: Δ = 0.01 [−0.01, 0.03]), while response times were faster (MPH > PLC: Δ = −0.02 [−0.07, 0.01]). Response times were less variable on MPH compared to placebo (MPH > PLC: Δ = −0.03 [−0.05, −0.01]). Results were qualitatively unchanged in the adherence-filtered sensitivity analysis (Supplementary Figure S7 and Supplementary Table S4).

### Drift diffusion model

Combined response time and choice data from the value-based decision-making task were analyzed with the DDM. $\hat{R}$ values for all chains in the model were between 1 and 1.02, indicating that the chains converged successfully (Gelman & Rubin, 1992). Posterior predictive checks indicated that the model captured key behavioral structure in the task: (i) the probability of accepting increased as benefit values increased and decreased as cost values increased, and (ii) the model reproduced the observed acceptance patterns across the joint benefit–cost space (Supplementary Figures S3–S6). In addition, posterior-predicted accuracy and response times closely matched the observed group-level summaries (Supplementary Table S2), supporting that the model also regenerates between-group differences in performance. Finally, PSIS-LOO favored the reported separate-sensitivity model over the net-value model (ΔELPD = 23.12, SE = 8.68; Supplementary Table S3).

#### Effect of ADHD on decision parameters

**Figure 3.**
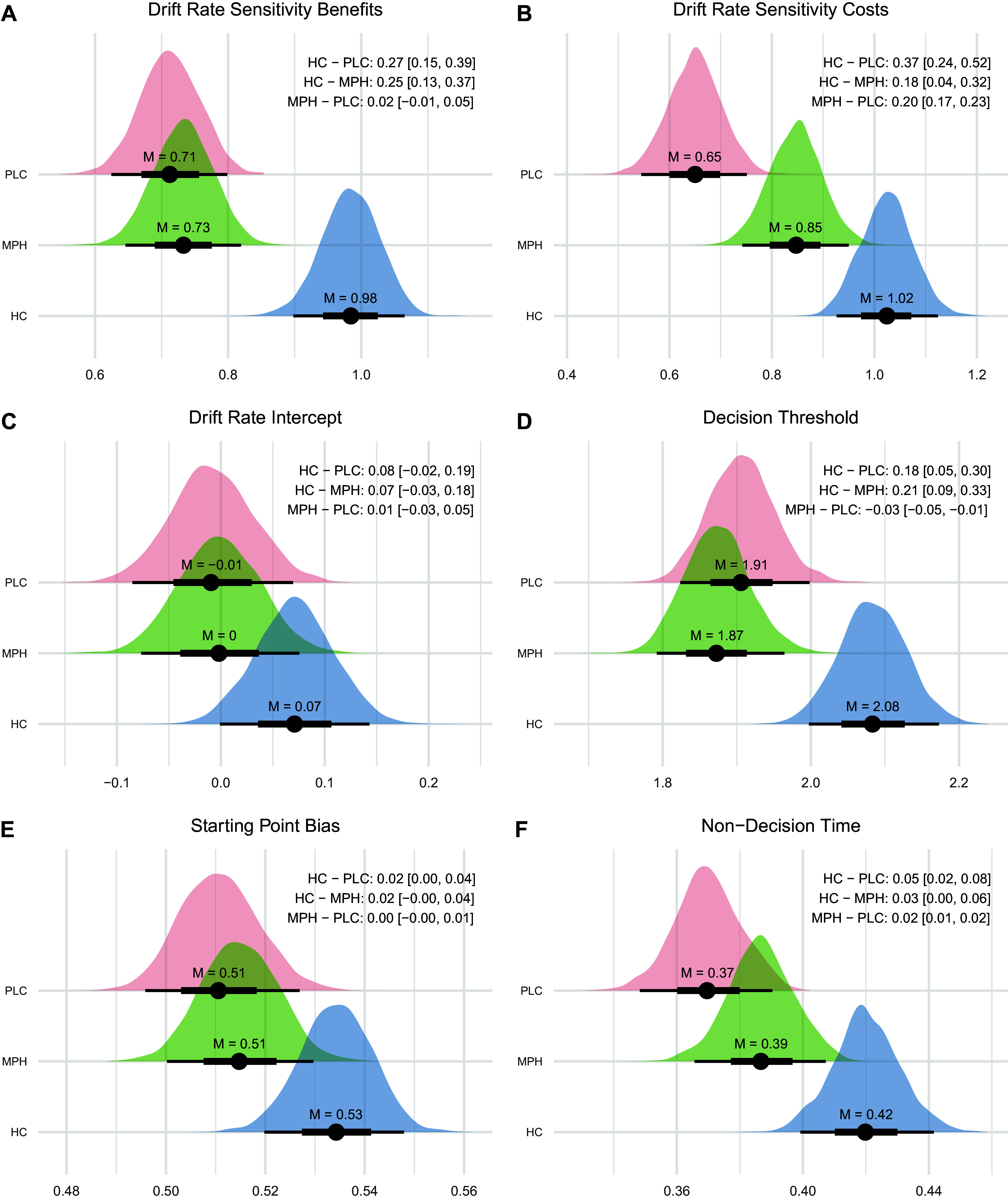
Posterior distributions of drift diffusion results (mITT sample). Estimated posterior distributions for mean impact of benefit (a), cost (b), and intercept (c) values onto drift rate, decision threshold (d), starting point bias (e), and nondecision time (f) for each group with pairwise contrasts reported as posterior mean differences (Δ) with 95% credible intervals (CrIs). Points indicate posterior medians, and thick/thin intervals indicate 66%/95% CrIs. The CrIs include between-participant variation, so that within comparisons can show clear effects even when CrI intervals for groups overlap. *Note*: HC, healthy controls; MPH, methylphenidate; PLC, placebo. Figure 3. long description.

In line with our predictions, ADHD participants on placebo had reduced sensitivity to benefit (HC > PLC: Δ = 0.27 95% credible interval [0.15, 0.39]) and cost (HC > PLC: Δ = 0.37 [0.24, 0.52]) stimuli, where reduced sensitivity yields smaller drift rates and slower evidence accumulation resulting in slower and less accurate decisions (Figure 3, Table 2). Sensitivity to cost and benefit stimuli was also reduced compared to controls when participants were on MPH (HC > MPH: Δ(benefit) = 0.25 [0.13, 0.37], HC > MPH: Δ(|cost|) = 0.18 [0.04, 0.32]). ADHD participants had narrower decision thresholds than controls both on (HC > MPH: Δ = 0.21 [0.09, 0.33]) and off (HC > PLC: Δ = 0.18 [0.05, 0.3]) medication, which, combined with slower drift rate, indicates premature responding. Nondecision times, accounting for time spent on visual encoding of stimuli and motor response, were faster for ADHD participants on (HC > MPH: Δ = 0.03 [0.0, 0.06]) and off (HC > PLC: Δ = 0.05 [0.02, 0.08]) MPH compared to controls. Starting points were estimated to be biased toward accepting offers in all groups, and this effect was stronger in control participants compared to ADHD participants on (HC > MPH: Δ = 0.02 [−0.0, 0.04]) and off (HC > PLC: Δ = 0.02 [0.0, 0.04]) MPH.

#### Effect of MPH on decision parameters

MPH improved drift rate for sensitivity to cost stimuli (MPH > PLC: Δ = 0.2 [0.17, 0.23]) more than sensitivity to benefit stimuli (MPH > PLC: Δ = 0.02 [−0.01, 0.05]). The contrast in MPH effect for cost versus benefit was strong (Δ(|cost|) > Δ(benefit)= 0.18 [0.13, 0.22]). MPH resulted in a further reduction in decision threshold (MPH > PLC: Δ = −0.03 [−0.05, −0.01]). Nondecision times increased following MPH (MPH > PLC: Δ = 0.02 [0.01, 0.02]). Administration of MPH also increased the starting point bias toward the accept threshold (MPH > PLC: Δ = 0.0 [−0.0, 0.01]). Results were qualitatively unchanged in the adherence-filtered sensitivity analysis (Supplementary Figure S8; Supplementary Table S4).

#### Sensitivity to cost versus benefit

**Figure 4.**
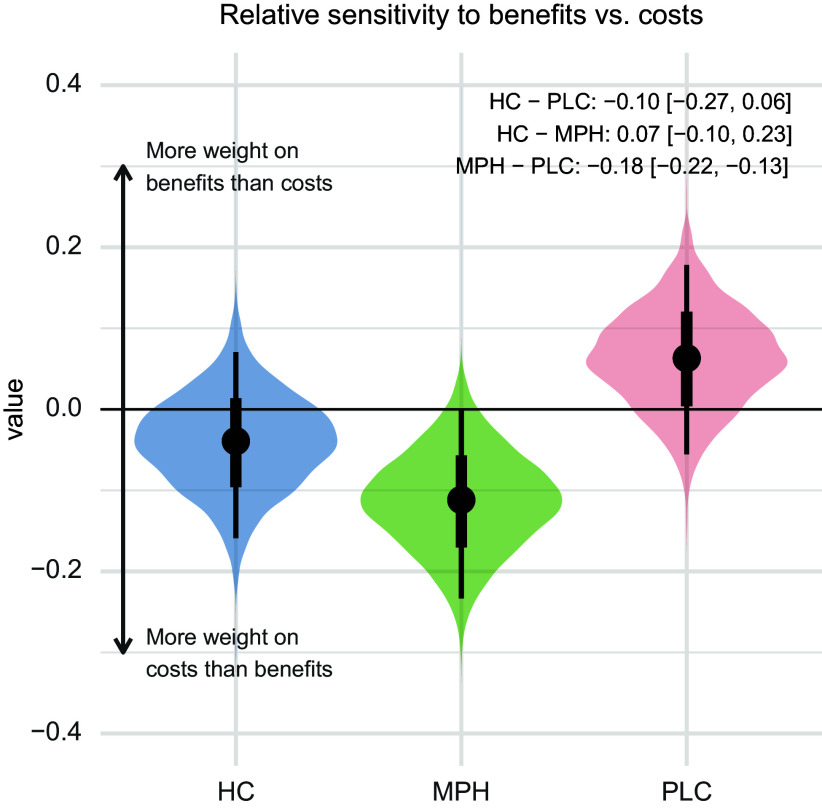
Cost–benefit balance on drift rate across groups (mITT sample). Posterior distributions of con trasts for the weight in benefit over cost sensitivity for each group, with pairwise contrasts reported as posterior mean differences (Δ) with 95% credible intervals (CrIs). Points indicate posterior medians, and thick/thin intervals indicate 66%/95% CrIs. Figure 4. long description.

A comparison of cost and benefit sensitivities in their influence on drift rate (Figure 4) across groups revealed that healthy controls were more sensitive to costs than to benefits, whereas ADHD participants off medication were more sensitive to benefits than to costs. Contrasts on these different measures revealed that MPH altered the balance between sensitivity to costs versus benefits in ADHD participants (MPH > PLC: Δ = −0.18 [−0.22, −0.13]). Results were qualitatively unchanged in the adherence-filtered sensitivity analysis (Supplementary Figure S9).

### Individual-differences analyses

We next examined whether individual differences in symptom severity, prescribed MPH dose, and serum ritalinic acid within ADHD participants were associated with variation in the estimated DDM parameters. These analyses were restricted to the adherence-filtered ADHD sample to minimize confounding from protocol nonadherence (elevated ritalinic acid during the placebo session) and because blood- and dose-based predictors are only meaningful when medication status is well-defined.

#### Symptom severity

We estimated the association of symptom severity, measured with WURS (Ward et al., 1993), to individual decision parameters in ADHD on and off medication (see Supplementary Table S1 for estimated group distributions and contrasts). In line with findings of reduced cost sensitivity in ADHD, we found that off medication, participants with more severe symptoms had reduced sensitivity to cost stimuli (β_wurs_ = −0.36 [−0.62, −0.1]).

#### Prescribed MPH dose

Next, we assessed whether prescribed dosage was associated with individual decision parameters when ADHD participants were on MPH. Higher prescribed dosage was associated with stronger sensitivity to costs (β_dose_ = 0.31 [0.05, 0.56]), negative drift rate intercept indicating a priori tendency of rejecting offers (β_dose_= −0.32 [−0.58, −0.06]), and higher decision threshold (β_dose_ = 0.38 [0.13, 0.62]) (see Supplementary Table S1 for estimated group distributions and contrasts).

#### Serum ritalinic acid

Finally, exploratory analyses tested whether serum ritalinic acid measured during the MPH session was associated with individual DDM parameter estimates. Across parameters, posterior mean slopes were small, and 95% credible intervals included zero, providing no strong evidence for exposure–response relationships in this sample (Supplementary Table S1).

## Discussion

To investigate how MPH alters mechanisms underlying cost-benefit decision-making in adult ADHD, we jointly analyzed choice and response times with the drift diffusion model (DDM). Impaired decision-making in the ADHD group was driven by reduced drift rates and narrower decision boundaries. Thus, ADHD participants accumulated evidence less efficiently and responded before having accumulated sufficient information. Administration of MPH selectively improved drift rates in the ADHD group by increasing sensitivity to information regarding the cost of offers. Further, administration of MPH caused an effect of increased narrowing of decision thresholds. MPH also resulted in slower nondecision times. Finally, we found that higher symptom severity in ADHD participants was associated with lower sensitivity to cost, while medication dose was associated with stronger cost sensitivity and higher decision threshold. Key medication effects were consistent in direction and magnitude in the adherence-filtered sensitivity analysis (see Supplementary Material), supporting robustness to protocol-adherence exclusions.

### Decision-making processes in ADHD

We have previously derived predictions for the effect of ADHD on DDM parameters from neurobiological theories of ADHD (Ziegler et al., 2016). The theories predicted reduced drift rate and narrower decision thresholds as the drivers of deficient decision-making in ADHD. Our results are in line with these predictions as both drift rate and decision threshold were reduced in ADHD compared to controls. An extension of predicting catecholamine-dependent inefficient evidence accumulation and premature responding in ADHD is that increased catecholaminergic signaling should ameliorate these deficiencies. The predictions on the effect of stimulant medication were only partly confirmed; MPH partly ameliorated drift rate, but only through increasing sensitivity to costs, and decision thresholds were found to be further decreased compared to controls.

Computational modeling studies of decision-making in ADHD reveal a consistent effect of impaired evidence accumulation across simple perceptual tasks and more cognitively demanding tasks (Fosco et al., 2017; Karalunas & Huang-Pollock, 2013; Karalunas, Huang-Pollock, & Nigg, 2012; Metin et al., 2013; Salum et al., 2014a, 2014b; Weigard & Huang‐Pollock, 2014). This effect could be driven by changes in catecholaminergic signaling, given the implication of DA and NE in decision-making processes. Dopamine improves the signal-to-noise ratio of cortical representations (Durstewitz, 2006), is involved in striatal filtering of cortical input (Nicola, Hopf, & Hjelmstad, 2004), and in maintaining decision values in working memory (Frank et al., 2007), while NE increases task-related activity and facilitates action selection (Berridge & Waterhouse, 2003). Together, these processes should all contribute to reducing noise in the decision process, translating to slower drift rates under lower levels of DA and NE in ADHD, and a positive effect of stimulant medication. Studies have found that MPH increases drift rate under perceptual decision-making in children with ADHD (Fosco et al., 2017) and healthy adults (Beste et al., 2018), and improves drift rate in adult ADHD participants during instrumental learning (Pedersen, Frank, & Biele, 2017), supporting the assumption that catecholamines modulate neural evidence accumulation (but see Mandali et al. [2021] for null-effect on drift rate).

In our study, MPH selectively enhanced sensitivity to costs in driving the decision to accept or reject offers. Sensitivity to costs was also negatively associated with symptom severity in ADHD participants off medication, and prescribed dosage was positively associated with cost sensitivity when ADHD participants received medication. While healthy controls and ADHD participants on medication showed stronger sensitivity to costs compared to benefits, ADHD participants off medication showed stronger sensitivity to benefits than costs. These results suggest that ADHD is associated with alterations in balancing how cost-benefit information guides decisions. Although we modeled the main-phase choices as value-based evidence accumulation, task performance depends on learned stimulus–value associations acquired during training and on subsequent retrieval and integration of benefit and cost information. MPH could therefore influence estimated cost sensitivity indirectly by improving the fidelity of learned value representations, by enhancing working-memory–supported integration of cost and benefit cues, or by altering decision formation more directly via catecholaminergic modulation of evidence accumulation. Other studies have found that ADHD is associated with decreased sensitivity to losses in temporal discounting and gambling (Luman et al., 2008; Tanaka et al., 2018). Together, these results indicate that impaired real-life decisions could partly result from reduced consideration of the negative aspects of actions. This effect could be contrasted to that of reduced sensitivity to risk as a cause of poorer real-life decisions in ADHD (Barkley et al., 2002; Faregh & Derevensky, 2011; Flory et al., 2006; Lee et al., 2011; Molina & Pelham, 2003; Sarver et al., 2014). Interestingly, recent studies (Dekkers et al., 2021; Pollak et al., 2016; Pollak, Shalit, & Aran, 2018; Sørensen et al., 2017) have found participants with ADHD to not be more risk-prone than their peers if value and risk are decoupled. Instead, ADHD was found to be associated with suboptimal decision making (i.e. choosing options with lower value). Future studies could investigate the separate contribution of sensitivity to costs and benefits while also manipulating risk to directly compare their contributions to suboptimal decisions.

The effect of ADHD and catecholamines on decision thresholds is less clear. Meta-analyses have not found conclusive effects of ADHD on decision boundaries (Karalunas et al., 2014; Mowinckel et al., 2015). Mulder and colleagues, on the other hand, found impairments in optimally adjusting the tradeoff of speed and accuracy in ADHD (Mulder et al., 2010). Despite striatal involvement in setting decision thresholds (Bogacz, Wagenmakers, Forstmann, & Nieuwenhuis, 2010; Forstmann et al., 2008), giving rise to assumptions of the involvement of DA in this process, neither MPH nor the dopamine receptor agonist Bromocriptine altered decision thresholds in healthy controls (Beste et al., 2018; Winkel et al., 2012). However, stimulant medication increased thresholds for ADHD participants during instrumental learning (Pedersen et al., 2017). In the current study, we found that MPH slightly reduced thresholds, which is inconsistent with predictions drawn from neurobiological theories of ADHD (Ziegler et al., 2016). One possible explanation is that a reduced threshold is a byproduct of an increased drift rate. Because ADHD participants accumulated evidence faster on than off MPH, they could reduce the decision threshold to ensure responding within the task response window while still maintaining a satisfactory level of accuracy. Indeed, we observed a higher rate of nonresponses in the placebo condition (PLC: 3.4%) compared to when ADHD participants were on medication (MPH: 1.3%), which may reflect insufficient evidence accumulation to reach a decision threshold within the time window in these trials. An implication of this explanation is that the threshold would not be reduced under MPH if participants were told to focus only on accuracy and/or were given a longer response window. In line with the potential benefits of MPH on decision thresholds, we found that higher MPH dosage was associated with wider decision thresholds. More studies are needed to understand under which conditions ADHD and DA are related to the optimization of speed-accuracy tradeoffs.

Administration of the MPH led to an unexpected increase in nondecision time. Nondecision time captures stimulus encoding and motor response. Given the role of DA in improving the motor system (Birkmayer & Hornykiewicz, 1998), it could be that the effect observed here is associated with stimulus encoding (Huang-Pollock et al., 2017). A potential mechanism could be that spending more time on stimulus encoding improves the accuracy of the stimulus representation, which would contribute to the higher estimated drift rate we observed.

### Limitations

Several task features beyond value sensitivity could contribute to the observed medication effects. First, MPH could influence learning during the training phase (Montague, Dayan, & Sejnowski, 1996) and thereby alter the precision of stimulus–value representations used in the main phase. While overall training accuracy did not differ between medication conditions, this does not rule out differences in learning dynamics (e.g. speed of acquisition or trial-to-trial variability) that could carry over to later choices. Second, the main phase requires retrieving the benefit and cost values associated with two stimulus features and integrating them to guide an accept/reject decision. MPH may improve working memory and value retrieval, potentially increasing the consistency with which costs are represented and incorporated into the decision process. Third, some participants may rely on approximate mental arithmetic or explicit comparison strategies (e.g. benefit minus cost), which could also be modulated by stimulants.

Future studies could disentangle these mechanisms by manipulating (i) the amount of training (overtraining vs. minimal training, or explicit value instruction without learning), (ii) working-memory demands during the main phase (dual-task load, delay, or set size), and (iii) cue integration (simultaneous vs. sequential presentation of cost and benefit cues). Pharmacological designs that more selectively target dopamine versus noradrenaline systems, combined with behavioral and physiological measures of evidence accumulation, could further test whether catecholaminergic modulation acts primarily on learning, value representation, or the decision process itself.

We excluded ADHD participants with major comorbid disorders to specifically investigate the effects of ADHD. It is therefore unclear to what extent our results generalize to ADHD with severe comorbid disorders. Lastly, we cannot exclude potential nocebo effects, as ADHD participants could have detected whether they received medication.

## Conclusions

ADHD participants made value-based choices with reduced sensitivity to cost and benefit stimuli and narrower thresholds compared to controls. MPH selectively improved cost sensitivity, while benefit sensitivity was unchanged. Lower cost sensitivity was associated with higher symptom severity, and greater improvement in cost sensitivity by MPH was associated with higher prescribed dosage. Together, the findings suggest MPH helps restore cost–benefit balance in ADHD, potentially informing everyday decision-making.

## Supporting information

10.1017/S0033291726104954.sm001 Pedersen et al. supplementary material Pedersen et al. supplementary material

## Data Availability

Custom code used in this study and synthetic data are available at https://osf.io/5h932/.

## Supplementary material

The supplementary material for this article can be found at http://doi.org/10.1017/S0033291726104954.

## Long descriptions

### Figure 1. Long description

Panel A, titled Value-based decision making task, shows three rows of trial sequences. The first row, Cost block training, shows a participant choosing between two colored blobs, receiving a checkmark and a negative numerical value like minus 8. The second row, Benefit block training, shows a choice between two geometric shapes, receiving an X and a positive value like 5. The third row, Main phase, shows a fixation cross for 2.5 to 9.5 seconds in M R I or 0.5 seconds in B E H, followed by a red cross for 0.5 seconds, and finally a colored shape stimulus for 2.5 seconds. Panel B, titled Drift Diffusion model, is a line graph. A horizontal line labeled Starting point begins on the left, followed by a flat segment labeled N D T. A jagged, noisy line then fluctuates up and down, representing evidence accumulation. The graph is bounded by a blue horizontal line at the top labeled Accept threshold and a green horizontal line at the bottom labeled Reject threshold. The noisy line eventually hits the Accept threshold. Below the path is the equation: Drift rate sub t equals beta sub benefit times X sub benefit open parenthesis t close parenthesis plus beta sub cost times X sub cost open parenthesis t close parenthesis.

Navigate back to Figure 1..

### Table 1. Long description

The table is organized into five columns: Characteristic, A D H D Mean, A D H D S D, Controls Mean, and Controls S D.

* Sample Size N (male/female): A D H D group has 57 (20 male, 37 female); Controls group has 60 (19 male, 41 female).

* Education in years: A D H D Mean 12 (S D 1.8); Controls Mean 14.7 (S D 2.2).

* Age in years: A D H D Mean 27.9 (S D 6); Controls Mean 27.4 (S D 5.7).

* A S R S (Adult A D H D Self-Report Scales): A D H D Mean 12.3 (S D 4.9); Controls Mean 6.9 (S D 2.9).

* W A I S (Wechsler Adult Intelligence Scale) similarities: A D H D Mean 10.3 (S D 3.4); Controls Mean 12.9 (S D 3).

* W A I S matrices: A D H D Mean 10.7 (S D 3.5); Controls Mean 12.6 (S D 3.1).

* Test interval in days: A D H D Mean 29.6 (S D 12.2); Controls Mean 28.4 (S D 10.5).

Navigate back to Table 1..

### Table 2. Long description

The table contains nine behavioral and modeling parameters across six comparative columns: H C, M P H, P L C, H C vs. P L C, H C vs. M P H, and M P H vs. P L C. Values are presented as means with 95 percent credible intervals in brackets.

* Accuracy: H C 0.91, M P H 0.86, P L C 0.85. The largest difference is H C vs. P L C at 0.07.

* Mean R T: H C 1.02, M P H 0.95, P L C 0.97.

* S D R T: H C 0.36, M P H 0.33, P L C 0.36.

* Starting point bias: H C 0.53, M P H 0.51, P L C 0.51.

* Decision threshold: H C 2.08, M P H 1.87, P L C 1.91. H C vs. M P H shows a difference of 0.21.

* Non-decision time: H C 0.42, M P H 0.39, P L C 0.37.

* Cost sensitivity: H C 1.02, M P H 0.85, P L C 0.65. H C vs. P L C shows the highest difference at 0.37.

* Benefit sensitivity: H C 0.98, M P H 0.73, P L C 0.71.

* Drift rate intercept: H C 0.07, M P H minus 0.00, P L C minus 0.01.

Note: H C stands for healthy control, M P H for methylphenidate, and P L C for placebo.

Navigate back to Table 2..

### Figure 2. Long description

A multi-panel figure with three vertical panels labeled A, B, and C. Each panel contains three stacked density plots for groups P L C (pink), M P H (green), and H C (blue), with median points (M) and horizontal credible interval bars.

Panel A: Accuracy. The x-axis is Predicted Accuracy from 0.80 to 0.95.

* P L C: M equals 0.85.

* M P H: M equals 0.86.

* H C: M equals 0.91.

Pairwise contrasts: H C minus P L C is 0.07 [0.04, 0.10]; H C minus M P H is 0.06 [0.03, 0.09]; M P H minus P L C is 0.01 [-0.01, 0.03].

Panel B: Mean R T. The x-axis is Mean R T in seconds from 0.9 to 1.1.

* P L C: M equals 0.97.

* M P H: M equals 0.95.

* H C: M equals 1.02.

Pairwise contrasts: H C minus P L C is 0.05 [-0.02, 0.12]; H C minus M P H is 0.07 [0.00, 0.14]; M P H minus P L C is -0.02 [-0.07, 0.01].

Panel C: R T Variability. The x-axis is R T S D in seconds from 0.300 to 0.400.

* P L C: M equals 0.36.

* M P H: M equals 0.33.

* H C: M equals 0.36.

Pairwise contrasts: H C minus P L C is -0.00 [-0.03, 0.02]; H C minus M P H is 0.03 [0.00, 0.05]; M P H minus P L C is -0.03 [-0.05, -0.01].

Navigate back to Figure 2..

### Figure 3. Long description

A multi-panel figure with six panels labeled A through F. Each panel displays three stacked posterior distribution curves (ridge plots) for three groups: P L C (pink, top), M P H (green, middle), and H C (blue, bottom). Each curve includes a black dot for the median and horizontal bars for 66 percent and 95 percent credible intervals. Text in the top right of each panel lists pairwise differences with 95 percent credible intervals.

* Panel A: Drift Rate Sensitivity Benefits. Medians are P L C 0.71, M P H 0.73, and H C 0.98. H C is significantly higher than both P L C and M P H.

* Panel B: Drift Rate Sensitivity Costs. Medians are P L C 0.65, M P H 0.85, and H C 1.02. All three groups show distinct, non-overlapping peaks.

* Panel C: Drift Rate Intercept. Medians are P L C negative 0.01, M P H 0, and H C 0.07. H C is shifted slightly to the right.

* Panel D: Decision Threshold. Medians are P L C 1.91, M P H 1.87, and H C 2.08. H C shows a higher threshold than the other two groups.

* Panel E: Starting Point Bias. Medians are P L C 0.51, M P H 0.51, and H C 0.53. Distributions are highly overlapping but H C is slightly higher.

* Panel F: Non-Decision Time. Medians are P L C 0.37, M P H 0.39, and H C 0.42. There is a clear step-wise increase from P L C to M P H to H C.

Navigate back to Figure 3..

### Figure 4. Long description

A violin plot titled Relative sensitivity to benefits vs. costs. The y-axis is labeled value and ranges from negative 0.4 to 0.4, with a horizontal zero line. A vertical double-headed arrow on the left indicates that values above zero represent more weight on benefits than costs, while values below zero represent more weight on costs than benefits.

Three groups are plotted along the x-axis:

1. H C (blue violin): The median point is slightly below zero. The distribution is symmetrical around the median with thick and thin vertical error bars.

2. M P H (green violin): The median point is lower than the H C group, centered around negative 0.1.

3. P L C (pink violin): The median point is above zero, centered around 0.07.

In the top right corner, pairwise contrasts are listed:

* H C minus P L C: negative 0.10 with a 95 percent credible interval of negative 0.27 to 0.06.

* H C minus M P H: 0.07 with a 95 percent credible interval of negative 0.10 to 0.23.

* M P H minus P L C: negative 0.18 with a 95 percent credible interval of negative 0.22 to negative 0.13.

Navigate back to Figure 4..
