# Supplementary material for: Selective enhancement of cost sensitivity by methylphenidate in adult ADHD: a randomized placebo-controlled trial
Source: Psychol Med. 2026 Jul 28;56:e234. doi: 10.1017/S0033291726104954 (PMC13439260; doi:10.1017/S0033291726104954)
Supplement: Supplementary file 1 [file S0033291726104954sup001.docx]

## Supplementary Material

### S1 – Exclusion criteria

- Treatment with the following groups of pharmacological agents will be
  considered as exclusion criteria for participation:
  - Antidepressants (MOA-inhibitors, Tricyclic antidepressants, SSRIs)
  - Antipsychotics (both first and second generation)
  - Anxiolytics/hypnotics (benzodiazepines, barbiturates)
  - Opiates
- History of alcohol or drug abuse.
- History of moderate to severe head injury.
- Individuals with intelligence (mean age scaled WAIS-IV score below 4 (Raven’s Matrices and Verbal similarities), corresponding to 2 standard deviations of the normal population).
- Major psychiatric comorbidity (i.e. psychosis, active suicidal ideation or acute exacerbation of other psychiatric condition in need of immediate treatment).
- Epilepsy
- History of severe memory loss
- Under treatment for metabolic disorders
- Severe primary sensory loss
- MRI specific criteria: contraindications for MRI (i.e. metallic or circuit-containing implants, severe claustrophobia)

### S2 - Diagnostic procedures

The ADHD diagnosis was assessed prior to study recruitment by two board certified psychiatrists, and established by a multistage and multisource procedure according to DSM-IV-TR criteria (American Psychiatric Association, 1994), with the Norwegian version of the structured Diagnostic Interview for ADHD in adults, second edition (DIVA 2.0; Kooij & Francken, 2010), and other informant sources (i.e. parents, siblings, significant-others etc.). Co-morbid psychiatric disorders were screened for by the MINI International Neuropsychiatric Interview Plus (M.I.N.I.-Plus; Sheehan et al., 1998). Only patients with established diagnosis of ADHD were asked to participate in the study.

After completion of the DIVA 2.0, participants needed to: 1) have at least six out of nine DSM-IV symptoms of inattention and/or hyperactivity/impulsivity in childhood, 2) have at least six out of the same nine DSM-IV symptoms for the last 6 months prior to examination, 3) describe a chronic course from childhood to adulthood without any indication of ADHD-free periods, 4) have five out of nine symptom criteria for each symptom domain in adulthood given they had met full symptom criteria in childhood (DSM-IV: ADHD Not Otherwise Specified), 5) have current ADHD symptoms that cause clinically significant impairment in social, educational, or occupational functioning.

### S3 - Pill-administration and randomization procedures

A random number generator was used through Matlab® to allocate 50% to first MPH then placebo condition and 50% to first placebo then MPH condition. This allocation was performed for blocks of 10 successive patients for altogether 200 patients. This block-wise randomization was chosen to ensure approximately equal size of each group (first placebo/first MPH) independent of how many patients could be recruited into the study. 100 such randomization lists were sent to Kragerø Tablettproduksjon a/s (pharmaceutical company) for placebo-production and medicament blinding, where one list was chosen and used to randomize patients into conditions. Study personnel and participants were blind to the drug conditions. The data were un-blinded after the last participant completed the study. The Ritalin™ capsules were over-encapsulated with CapsuGel DBcaps® AAel (Swedish Orange Opaque), and so were the corresponding placebo capsules. The MPH and placebo tablets were equal in color, shape and size. All pills were taken straight out of a light-isolating box, giving neither participant nor experimenter the possibility to identify the pills. No study personnel were involved in the blinding procedure or randomization.

### S4 - Recruitment dates

The first participant in the MR cohort was recruited on Sept. 30, 2013, and the last participant was completed on June 18, 2014. Recruitment and follow-up period of the fMRI-arm was terminated early due to scanner change, where we did not wish to introduce further noise to the analysis by collecting data from two separate scanners. The first participant in the behavioral cohort was recruited on Oct. 17, 2013, and the last participant was completed due to end of data collection phase on July 10, 2015.

### S5 - Blood tests and analysis

#### Blood tests

We collected blood samples from consenting ADHD patients both as a control that cessation of medication was upheld and to estimate effects of ritalinic acid, a metabolite of MPH, and the protein prolactin, acting as an indirect, inversely related measure of DA levels (Ben-Jonathan & Hnasko, 2001) on performance in the decision making task. Blood samples were collected in red 10ml Becton Dickinson Vacutainer® tubes (with clot activation) on average 2 hours (36 min SD) after intake of pills. Samples rested a minimum of 30 minutes (maximum 1 hour) before centrifuging at 1,100g for 10 minutes. Serum was extracted and frozen at –20°C within 12 hours from collection. Analysis of ritalinic acid was performed at The Center for Psychopharmacology, Diakonhjemmet Hospital, Oslo, Norway, while analysis of prolactin levels was performed by Fürst laboratory.

#### Blood analysis

Serum concentration of ritalinic acid was measured by an ultraperformance liquid chromatography-mass spectrometry (UPLC-MS/MS) method developed for routine therapeutic drug monitoring analyses. Serum samples were prepared with protein precipitation using acetonitrile. Analysis was performed using an Acquity UPLC linked to a Micromass Quattro Micro Tandem MS detector (Waters, Milford, MA, USA). Chromatographic separation was achieved using an Acquity UPLC BEH shield RP18 column (1.7µm, 1x100 mm; Waters) with gradient elution at 40 °C with a mix of ammonium acetate buffer (pH 4.8) and acetonitrile (20–50%) as mobile phase. The retention time was 0.62 min. Detection with multiple reaction monitoring was performed at m/z 220-84 transitions. Deuterated ritalinic acid was used as internal standard (m/z 230-93, retention time 0.61 min). The calibration curve was 200-4000 nM.

Levels of prolactin were measured with the ADVIA Centaur XPT System (Siemens Medical Solutions Diagnostics). ADVIA Centaur Prolactin assay is a two-step sandwich immunoassay using direct chemical illuminometric technology, using constant amounts of two antibodies. The first antibody in the Lite Reagent is a polyclonal anti-prolactin antibody from goat labeled with acridinium. The second antibody in the solid phase is a monoclonal anti-prolactin mouse antibody covalently bound to paramagnetic particles. Prolactin in the patient sample bind to the acridinium-labeled antibody. The monoclonal mouse anti-prolactin antibody, bound to paramagnetic particles is added and the sample is incubated before the bound separated from the unbound by magnetic separation. After addition of acid and base takes place chemiluminescence reaction. Flashes of light detected in the luminometer as relative light units (RLU). A direct relationship exists between the amount of prolactin present in the patient sample and the amount of relative light units that are detected by the system.

Table S1. Summary associations of individual decision parameters to symptom severity and prescribed dosage.

| **dependent variable** | **parameter** | **term** | **estimate** | **2.5 %** | **97.5 %** |
| --- | --- | --- | --- | --- | --- |
| Symptom Severity |  |  |  |  |  |
|  | Starting Point Bias | MPH | -0.001 | -0.371 | 0.372 |
|  | Starting Point Bias | dosage | 0.222 | -0.052 | 0.489 |
|  | Starting Point Bias | MPH:dosage | 0.002 | -0.38 | 0.394 |
|  | Decision Threshold | MPH | -0.001 | -0.386 | 0.386 |
|  | Decision Threshold | dosage | -0.072 | -0.355 | 0.207 |
|  | Decision Threshold | MPH:dosage | -0.001 | -0.393 | 0.389 |
|  | Non-Decision Time | MPH | 0.002 | -0.377 | 0.376 |
|  | Non-Decision Time | dosage | -0.176 | -0.457 | 0.103 |
|  | Non-Decision Time | MPH:dosage | -0.006 | -0.396 | 0.379 |
|  | Drift Rate Intercept | MPH | 0.001 | -0.384 | 0.383 |
|  | Drift Rate Intercept | dosage | 0.151 | -0.124 | 0.426 |
|  | Drift Rate Intercept | MPH:dosage | 0.003 | -0.387 | 0.394 |
|  | Cost sensitivity | MPH | 0.002 | -0.371 | 0.362 |
|  | Cost sensitivity | dosage | -0.362 | -0.623 | -0.102 |
|  | Cost sensitivity | MPH:dosage | -0.009 | -0.376 | 0.355 |
|  | Benefit sensitivity | MPH | 0 | -0.385 | 0.373 |
|  | Benefit sensitivity | dosage | -0.211 | -0.482 | 0.071 |
|  | Benefit sensitivity | MPH:dosage | -0.003 | -0.393 | 0.385 |
| Prescribed dosage |  |  |  |  |  |
|  | Starting Point Bias | dosage | -0.051 | -0.321 | 0.226 |
|  | Decision Threshold | dosage | 0.375 | 0.127 | 0.618 |
|  | Non-Decision Time | dosage | 0.074 | -0.202 | 0.349 |
|  | Drift Rate Intercept | dosage | -0.32 | -0.578 | -0.062 |
|  | Cost sensitivity | dosage | 0.308 | 0.051 | 0.561 |
|  | Benefit sensitivity | dosage | 0.069 | -0.201 | 0.339 |
| Blood levels (Ritalinic acid) | |  |  |  |  |
|  | Starting Point Bias | Ritalinic acid | 0.103 | -0.237 | 0.451 |
|  | Decision Threshold | Ritalinic acid | -0.231 | -0.581 | 0.122 |
|  | Non-Decision Time | Ritalinic acid | 0.091 | -0.317 | 0.509 |
|  | Drift Rate Intercept | Ritalinic acid | 0.176 | -0.205 | 0.55 |
|  | Cost sensitivity | Ritalinic acid | 0.111 | -0.235 | 0.46 |
|  | Benefit sensitivity | Ritalinic acid | -0.057 | -0.431 | 0.318 |

Table S2. Observed and predicted mean accuracy and response time across modified intention-to-treat (mITT) sample and adherence-filtered (AF) sample.

|  | **dataset** | **status** | **observed** | **predicted** | **difference** |
| --- | --- | --- | --- | --- | --- |
| **Accuracy** |  |  |  |  |  |
|  | AF | HC | 0.898 | 0.885 [0.881, 0.888] | -0.014 |
|  | AF | MPH | 0.833 | 0.835 [0.829, 0.842] | 0.002 |
|  | AF | PLC | 0.827 | 0.821 [0.815, 0.827] | -0.006 |
|  | mITT | HC | 0.898 | 0.885 [0.881, 0.889] | -0.014 |
|  | mITT | MPH | 0.831 | 0.834 [0.827, 0.840] | 0.002 |
|  | mITT | PLC | 0.827 | 0.817 [0.811, 0.824] | -0.011 |
| **RT** |  |  |  |  |  |
|  | AF | HC | 1.006 | 1.071 [1.063, 1.079] | 0.065 |
|  | AF | MPH | 0.947 | 1.001 [0.990, 1.011] | 0.054 |
|  | AF | PLC | 0.984 | 1.033 [1.020, 1.047] | 0.049 |
|  | mITT | HC | 1.006 | 1.071 [1.064, 1.079] | 0.065 |
|  | mITT | MPH | 0.938 | 0.993 [0.985, 1.002] | 0.056 |
|  | mITT | PLC | 0.976 | 1.023 [1.012, 1.032] | 0.046 |

Table S3. Model comparison. Leave-one-out (LOO) cross-validation metrics for model with separate sensitivity for costs and benefit (valence model) and single sensitivity for costs minus benefit (net-value model).Winning model is valence model.

| **model** | **method** | **elpd** | **SE** | **elpd_loo** | **SE** | **p_loo** | **SE** | **looic** | **SE** | **#pareto > 0.7** | **#pareto> 1.0** | **max_pareto_k** |
| --- | --- | --- | --- | --- | --- | --- | --- | --- | --- | --- | --- | --- |
| **valence** | base_psis | 0.00 | 0.00 | -32708 | 295.20 | 913.70 | 42.33 | 65416 | 590.40 | 66 | 31 | 1.78 |
| **net-value** | base_psis | -23.12 | 8.68 | -32731 | 295.41 | 912.63 | 42.94 | 65462 | 590.82 | 66 | 32 | 1.75 |

Table S4. Summary behavioral and modeling results (adherence-filtered sample). Mean and 95% credible intervals of posterior distributions.

| **Parameter** | **HC** | **MPH** | **PLC** | **HC vs. PLC** | **HC vs. MPH** | **MPH vs. PLC** |
| --- | --- | --- | --- | --- | --- | --- |
| Accuracy | 0.92 [0.9, 0.93] | 0.85 [0.83, 0.88] | 0.84 [0.82, 0.87] | 0.07 [0.04, 0.10] | 0.06 [0.03, 0.09] | 0.01 [-0.01, 0.03] |
| Mean RT | 1.02 [0.98, 1.07] | 0.96 [0.91, 1.01] | 0.98 [0.93, 1.04] | 0.04 [-0.03, 0.11] | 0.06 [-0.00, 0.13] | -0.03 [-0.07, 0.01] |
| SD RT | 0.36 [0.34, 0.38] | 0.33 [0.32, 0.35] | 0.36 [0.34, 0.38] | -0.00 [-0.03, 0.03] | 0.02 [-0.00, 0.05] | -0.02 [-0.04, -0.01] |
| Starting Point Bias | 0.53 [0.52, 0.55] | 0.52 [0.5, 0.53] | 0.51 [0.5, 0.53] | 0.02 [-0.00, 0.04] | 0.02 [-0.00, 0.04] | 0.00 [-0.00, 0.01] |
| Decision Threshold | 2.09 [2, 2.18] | 1.88 [1.79, 1.97] | 1.92 [1.83, 2] | 0.17 [0.04, 0.30] | 0.21 [0.08, 0.34] | -0.04 [-0.06, -0.01] |
| Non-Decision Time | 0.42 [0.4, 0.44] | 0.39 [0.37, 0.41] | 0.38 [0.36, 0.4] | 0.04 [0.01, 0.07] | 0.03 [-0.00, 0.06] | 0.01 [0.01, 0.02] |
| Cost Sensitivity | 1.02 [0.92, 1.12] | 0.83 [0.72, 0.94] | 0.65 [0.54, 0.76] | 0.37 [0.23, 0.51] | 0.20 [0.06, 0.33] | 0.17 [0.14, 0.21] |
| Benefit Sensitivity | 0.99 [0.9, 1.07] | 0.73 [0.64, 0.82] | 0.73 [0.64, 0.82] | 0.26 [0.14, 0.38] | 0.26 [0.13, 0.39] | 0.00 [-0.03, 0.03] |
| Drift rate Intercept | 0.07 [-0.01, 0.14] | -0.02 [-0.1, 0.07] | 0 [-0.08, 0.08] | 0.07 [-0.05, 0.18] | 0.09 [-0.03, 0.19] | -0.01 [-0.05, 0.03] |

HC = healthy control, MPH = methylphenidate, PLC = placebo.

**Fig S2. Training-phase screens.** During training trials participants saw two stimuli of same category and were instructed to choose largest benefit or smallest cost, depending on condition. Numeric values were not displayed on these screens; values were presented as feedback after each choice during training.

**Fig S3.** Posterior predictive RT distributions for each difficulty level across group, medication condition (within ADHD), session and cohort for the modified intention-to-treat (mITT) sample. Values on each column represent combined values of Benefit and cost stimuli. Correct choices would be to reject for combined negative values and accept for combined positive values. Density lines in black display observed results while density lines in blue represent generated results from parameter estimates. Positive RT distributions represent accept-choices, while reject-choices are set to be negative, by subtracting the reaction time from 0.

**Fig S4.** Posterior predictive heatmaps of mean observed and predicted probability to accept (1) and reject offers (0) across quartiles of costs and benefits in the modified intention-to-treat (mITT) sample.

**Fig S5.** Posterior predictive RT distributions for each difficulty level across group, medication condition (within ADHD), session and cohort for the modified adherence-filtered (AF) sample. Values on each column represent combined values of Benefit and cost stimuli. Correct choices would be to reject for combined negative values and accept for combined positive values. Density lines in black display observed results while density lines in blue represent generated results from parameter estimates. Positive RT distributions represent accept-choices, while reject-choices are set to be negative, by subtracting the reaction time from 0.

**Fig S6.** Posterior predictive heatmaps of mean observed and predicted probability to accept (1) and reject offers (0) across quartiles of costs and benefits in the adherence-filtered (AF) sample.

**Fig. S7.** Posterior distributions of choice and response time analyses (adherence-filtered sensitivity sample). Estimated posterior distributions for accuracy (A) response time (B) and response time (RT) variability (C) for each group, with pairwise contrasts reported as posterior mean differences (Δ) with 95% credible intervals (CrI). Points indicate posterior medians and thick/thin intervals indicate 66%/95% CrIs. The vertical CrIs include between-participant variation, so that within-comparisons can show clear effects even when CrI intervals for groups overlap. HC = healthy controls, MPH = methylphenidate, PLC = placebo, SD = standard deviation.

**Fig. S8.** Posterior distributions of drift diffusion results (adherence-filtered sensitivity sample). Estimated posterior distributions for mean impact of benefit (A), cost (B) and intercept (C) values onto drift rate, decision threshold (D), starting point bias (E) and non-decision time (F) for each group with pairwise contrasts reported as posterior mean differences (Δ) with 95% credible intervals (CrI). Points indicate posterior medians and thick/thin intervals indicate 66%/95% CrIs. The CrIIs include between-participant variation, so that within comparisons can show clear effects even when CrI intervals for groups overlap. HC = healthy controls, MPH = methylphenidate, PLC = placebo.

**Fig. S9.** Cost-benefit balance on drift rate across groups (adherence-filtered sensitivity sample). Posterior distributions of contrasts for the weight in benefit over cost sensitivity for each group, with pairwise contrasts reported as posterior mean differences (Δ) with 95% credible intervals (CrI). Points indicate posterior medians and thick/thin intervals indicate 66%/95% CrIs.

**Supplementary references:**

1.	Kruschke JK. Bayesian Estimation Supersedes the t Test. *Journal of Experimental Psychology: General*. 2012. doi:10.1037/a0029146.

2.	American Psychiatric Association. (1994). Diagnostic and statistical manual of mental disorders (4th ed.) (Vol. 29, pp. 229–239). Washington, DC: Author.

3.	Kooij JJS, Francken MH (2010) DIVA 2.0 Diagnostic Interview ADHD in Adults. DIVA Foundation.

4.	Sheehan DV, Lecrubier Y, Sheehan KH, Amorim P, Janavs J, Weiller E, Hergueta T, Baker R, Dunbar GC (1998) The Mini- International Neuropsychiatric Interview (MINI): the develop- ment and validation of a structured diagnostic psychiatric interview for DSM-IV and ICD-10. J Clin Psychiatry 59:22–33

5.	Ben-Jonathan, N., & Hnasko, R. (2001). Dopamine as a Prolactin (PRL) Inhibitor. *Endocrine Reviews*, *22*(6), 724–763. http://doi.org/10.1210/edrv.22.6.0451
